# Differential diagnosis between urticarial vasculitis and chronic spontaneous urticaria: An international Delphi survey

**DOI:** 10.1002/clt2.12305

**Published:** 2023-10-19

**Authors:** Karoline Krause, Hanna Bonnekoh, Jannis Jelden‐Thurm, Riccardo Asero, Ana Maria Gimenez‐Arnau, José C. Cardoso, Clive Grattan, Emek Kocatürk, Undine Lippert, Marcus Maurer, Martin Metz, Petra Staubach, Margarida Goncalo, Pavel Kolkhir

**Affiliations:** ^1^ Institute of Allergology Charité – Universitätsmedizin Berlin corporate member of Freie Universität Berlin Humboldt‐Universität zu Berlin Berlin Institute of Health Berlin Germany; ^2^ Fraunhofer Institute for Translational Medicine and Pharmacology ITMP Allergology and Immunology Berlin Germany; ^3^ Ambulatorio di Allergologia Clinica San Carlo Paderno Dugnano Italy; ^4^ Hospital del Mar and Research Institute of Barcelona Universitat Pompeu Fabra Barcelona Spain; ^5^ Department of Dermatology Centro Hospitalar e Universitário de Coimbra Coimbra Portugal; ^6^ Faculty of Medicine University of Coimbra Coimbra Portugal; ^7^ Guy's Hospital St John's Institute of Dermatology London UK; ^8^ Department of Dermatology Koç University School of Medicine Istanbul Turkey; ^9^ Department of Dermatology, Venereology and Allergology University Medical Center Göttingen Göttingen Germany; ^10^ Department of Dermatology University Medical Center Mainz Mainz Germany

**Keywords:** chronic spontaneous urticaria, chronic urticaria, consensus, diagnostic criteria, diagnosis, unmet needs, urticarial vasculitis

## Abstract

**Background:**

Urticarial vasculitis (UV) should be differentiated from chronic spontaneous urticaria (CSU) in patients initially presenting with recurrent wheals, although criteria for differential diagnosis remain ill‐defined.

**Objectives:**

To set the goals, define criteria and unmet needs in UV diagnosis and differential diagnosis with CSU, and explore the possibility of coexistence of both diseases.

**Methods:**

Thirteen experts experienced in UV research participated in a Delphi survey of European Academy of Allergy and Clinical Immunology taskforce. This Delphi survey involved three rounds of anonymous responses to *n* = 32 questions with the aim to aggregate the experts' opinions and to achieve consensus. Urticaria specialists (*n* = 130, most from Urticaria Centers of Reference and Excellence) evaluated the consensus statements and recommendations in the fourth and final round.

**Results:**

The panel agreed that essential criteria to guide a skin biopsy in patients with recurrent wheals should include at least one of the following features: wheal duration >24 h, bruising/postinflammatory hyperpigmentation, and systemic symptoms. Leukocytoclasia and fibrin deposits were identified as a minimum set of UV histological criteria. As agreed by the panel members, CSU and normocomplementemic UV (NUV) may coexist in some patients.

**Conclusions:**

The use of established criteria for the diagnosis and differential diagnosis of UV in patients with recurrent wheals can help guide the diagnostic approach and prompt earlier treatment. Further studies should investigate whether CSU and NUV are different entities or part of a disease spectrum.

## 1 INTRODUCTION

Urticarial vasculitis (UV) is a rare chronic inflammatory disease presenting with long‐lasting wheals with or without angioedema^1^ and the histopathological finding of leukocytoclastic vasculitis.^2^ In patients with UV, chronic wheals are often accompanied by burning and pain sensation. The pathogenesis of UV is unclear. Symptoms appear likely due to the deposition of immune complexes in the vessels and activation of the complement system.^3^, ^4^ Based on the consumption of complement as a marker of complement activation, normocomplementemic urticarial vasculitis (NUV, approx. 80% of UV patients) is distinguished from hypocomplementemic urticarial vasculitis (HUV).^5^ The clinical spectrum in UV patients varies and comprises systemic symptoms (especially in patients with HUV) such as musculoskeletal complaints, fever, fatigue, and other organ involvement.^6^ Treatment of UV patients is often difficult as no approved drugs exist and the use of off‐label drugs shows limited efficacy and/or severe side effects.^7^ In addition, the quality of life in UV patients is significantly impaired which is associated with long disease duration, high symptom burden, and high need for therapy.^1^

Chronic spontaneous urticaria (CSU) is the main differential diagnosis of UV.^2^, ^8^, ^9^ The clinical presentation of UV and some forms of severe CSU with systemic complaints displays considerable overlap,^10^ which can complicate the diagnosis of UV. In fact, up to 27% of patients initially presenting with CSU are diagnosed with UV over time.^11^ The use of skin biopsy, the gold standard for diagnosing UV, is limited by its invasiveness and lack of clear criteria for its indication.^2^ Leukocytoclasia, fibrin deposits within the lumen and inside the wall, and extravasated erythrocytes have been recently suggested as histologic criteria for UV, although their further validation is necessary.^12^ Besides histopathological findings, additional validated clinical criteria for the diagnosis of UV are needed. Currently, the management of UV patients is challenging for physicians, as no clinical guidelines, diagnostic criteria, or treatment algorithms exist.^13^

A subpopulation of CSU patients shows signs and symptoms characteristic of UV, for example, wheal duration >24 h (26%), bruising of the skin (9%), and systemic symptoms, for example, abdominal pain.^14^, ^15^ UV in some patients responded to therapy with omalizumab, an anti‐IgE monoclonal antibody.^16^ This suggests that UV and CSU could coexist in some patients and that CSU patients might develop UV over time and/or vice versa. For example, several skin biopsies performed in the same CSU patient over time showed both signs of urticaria and UV.^17^, ^18^

Therefore, several questions have been raised by research and experts: (1) Should CSU be differentiated from both forms of UV, that is, NUV and HUV, or one of them? (2) Can UV and CSU coexist in the same patient and if so, is there a time continuum or are they separate entities? (3) What are the criteria for differential diagnosis between UV and CSU? and (4) What clinical and laboratory criteria should be used as indicators to perform a skin biopsy in a patient with recurrent wheals?

To address these questions, we launched the Taskforce of the European Academy of Allergy and Clinical Immunology (EAACI) with the aim of bringing together experts from across Europe to highlight unmet needs in differential diagnosis between CSU and UV and to develop, standardize, and harmonize diagnostic criteria for UV. To the best of our knowledge, there are no previously reported attempts to reach a consensus on the diagnostic criteria for this rare and difficult‐to‐treat disease.

## 2 METHODS

### 2.1 Participant selection

The EAACI taskforce was established by KK (chair) and MG (secretary) in 2019 and with the further contribution of PK and HB, they represented the steering committee of this taskforce. The steering committee was responsible for the development of the project, overseeing the overall process, and managing the Delphi survey. In consultation with the chair and the secretary, 12 UV experts were selected for the invitation to the Delphi panel on the basis of involvement with scientific research and clinical care on UV. The final Delphi panel comprised 13 experts, the core expert group (all coauthors of this manuscript), and represented 6 countries (Germany, Italy, Spain, Portugal, United Kingdom, and Turkey). In order to gain a robust consensus, the consensus statements and recommendations were additionally evaluated by 130 worldwide urticaria specialists, mostly from Europe, the Americas, and Asia (*n* = 115 from Urticaria Centers of Reference and Excellence [UCAREs]^19^) (Table S1). No honoraria were paid for participation, but travel expenses and accommodation for two face‐to‐face meetings were covered by EAACI. Local ethics committee approval was not required as patients were not involved in this study.

### 2.2 Delphi process

**FIGURE 1:**
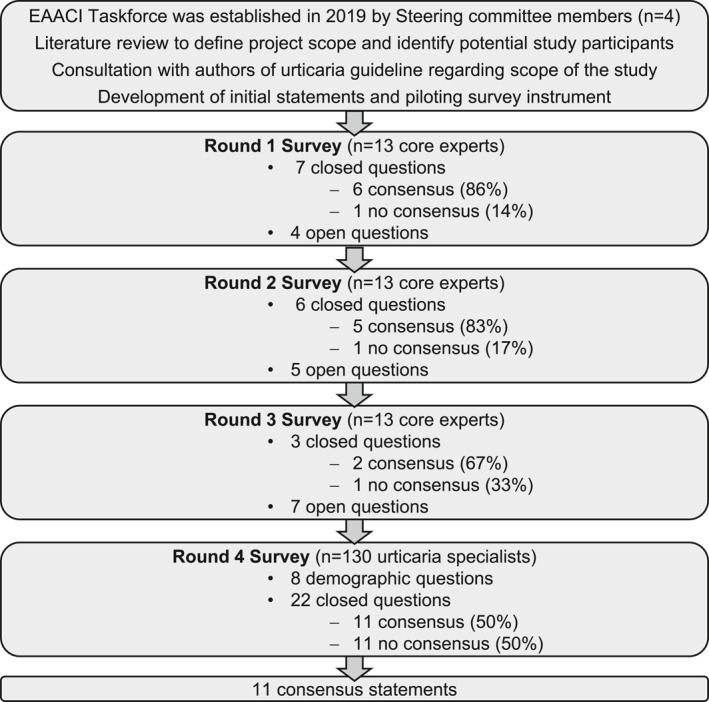
Flowchart of Delphi consensus process. A summary of the actions taken during each step of the Delphi consensus process is shown.

The Delphi process was performed between November 19, 2019, and December 12, 2022, similarly as described before (Figure 1, Figures S1–S4).^20^ The prolonged time frame of the process was due to the limitations of scientific interactions during the COVID‐19 pandemic. We conducted four anonymous electronic Delphi survey rounds. Consensus was defined a priori as agreement by at least 85% of respondents.^21^ Each survey round was scheduled for a period of 2 weeks. Google Forms software (Google, Mountain View, California, USA) and the online survey platform SurveyMonkey (SVMK Inc, San Mateo, California, USA) were used.

The Delphi process comprised three rounds within the core expert group of 13 members, with minor changes in the questions in each survey in order to increase the level of precision. In round 1, 11 questions were prepared by the steering committee in order to better define unmet needs and to establish consensus on the diagnostic approach of CSU versus UV. The results of round 1 of the Delphi process were discussed with the whole expert group at a taskforce meeting at the EAACI annual congress in Lisbon in June, 2019. After a new face‐to‐face meeting of the taskforce at the Global Urticaria Forum in Istanbul in 2019, a round 2 questionnaire was generated based on the results of round 1. The digital survey was shared with all experts and until November 2021, all 13 experts participated in the survey. In round 3, a questionnaire with 10 questions was developed based on the results of round 2. Until November 2022, all 13 experts participated in the survey. Based on the results of the third round, the expert opinions were aggregated and a set of expert consensus statements and questions was prepared by the steering committee and shared with the urticaria specialists. The survey was advertised at the Global Urticaria Forum in December 2022 in Berlin, Germany. Until December 12, 2022, 130 urticaria specialists participated in the fourth round of the Delphi process and completed the survey. The final set of consensus statements was prepared on the basis of ≥85% agreement of both the core expert group and urticaria specialists (Table 1). Consensus statements and recommendations were developed into a flowchart to support the diagnosis and differential diagnosis of UV, which was reviewed by the core expert group before receiving final approval.

**TABLE 1:** Summary of consensus statements after the final fourth round of the Delphi survey (% agreement core experts/urticaria specialists).^a^

Unmet needs in differential diagnosis between UV and CSU The definition of the diagnostic limits between chronic spontaneous urticaria (CSU) and urticarial vasculitis (UV) is an unmet need (100/88%) The existent definition of UV is wheals or erythematous plaques persisting for >24 h combined with the histopathologic findings of leukocytoclastic vasculitis (93/96%) The main diagnostic unmet need is to distinguish CSU from normocomplementemic UV (and not from HUV) (85/86%) The main diagnostic unmet need is to examine whether CSU and NUV are different entities or part of a disease spectrum presenting with wheals (100/94%) Further studies are necessary to better characterize the differences and similarities in CSU and UV patients (100/99%)
Coexistence of UV/NUV and CSU in same patients There is coexistence between CSU and NUV symptoms (e.g. transient wheals and long‐lasting lesions with bruising) in some patients at the same time (100/92%)
Clinical and laboratory criteria for differential diagnosis Long wheal duration (>24 h), bruising/postinflammatory hyperpigmentation, and systemic symptoms (e.g. abdominal pain, fever, and/or joint pain) are the main criteria for performing a skin biopsy in a CSU patient for differential diagnosis with urticarial vasculitis (100/92%). One or two of these findings but not necessarily all three (systemic symptoms, bruising/postinflammatory hyperpigmentation and/or long wheal duration) are enough to perform a skin biopsy in a CSU patient for differential diagnosis with urticarial vasculitis (100/87%). Leukocytoclasia and fibrin deposits on the vessel walls are required as a minimum set of criteria to establish a histopathologic diagnosis of UV (92/93%). If skin biopsy cannot be performed for any reason to differentiate between NUV and CSU, long wheal duration (>24 h) and bruising/purpura/postinflammatory hyperpigmentation are major criteria for possible diagnosis of NUV in a patient with recurrent wheals (85/86%). ANA and CRP are laboratory tests that should be performed in addition to skin biopsy in the case of occasional occurrence of long‐lasting lesions with transient purpura/bruising in a patient with recurrent wheals (92/91%).

^a^ Based on the three rounds of Delphi survey of core experts, all coauthors of this publication, 22 statements have been prepared with consensus 85% or more. One hundred thirty urticaria specialists, dermatologists and allergists, most from the Urticaria Centers of Reference and Excellence (UCARE) worldwide, were asked to agree or disagree with the consensus statements. Statements that did not reach consensus (<85%) for core experts or urticaria specialists are not shown and are included in Table S2.

## 3 RESULTS

### 3.1 Overview of the Delphi process

Most of the core experts were dermatologists with a median (IQR) of 20 (15–28) years of experience in the treatment of patients with UV and CSU. Among 130 urticaria specialists, 73 were allergists (56%), with a median (IQR) of 15 (10–25) years of experience in the treatment of patients with UV (Table S1).

We collected 100% of responses for rounds 1, 2, and 3 among the core experts, and a consensus was reached on 6 (86%), 5 (83%), and 2 (67%) of closed questions for rounds 1, 2, and 3.

Based on the experts' opinions, 22 consensus statements were prepared (Table S2). Urticaria specialists agreed with 11 (50%) of them. Final consensus statements based on ≥85% agreement of both experts and urticaria specialists are summarized in Table 1.

### 3.2 Consensus statements for unmet needs in differential diagnosis between UV and CSU

Respondents overwhelmingly agreed that the definition of the diagnostic limits between CSU and UV is an unmet need. Furthermore, 93% of core experts and 96% of urticaria specialists agreed that the existent definition of UV is wheals or erythematous plaques persisting for >24 h combined with the histopathologic findings of leukocytoclastic vasculitis. However, some experts have expressed doubts that all classic signs of leukocytoclastic vasculitis are seen in all UV patients.

From the agreed statements, there is an unmet need to differentiate between CSU and NUV (core experts/urticaria specialists: 85/86%), whereas hypocomplementemic UV (HUV) is considered by many urticaria specialists (though without consensus, 85%/80%) a different entity with no overlap with CSU.

Almost all core experts and urticaria specialists agreed that the main unmet diagnostic need is to examine whether CSU and NUV are different entities or part of a disease spectrum presenting with wheals. Finally, almost all core experts and urticaria specialists concluded (100/99%) that further studies are necessary to better characterize the differences and similarities between CSU and UV patients (Table 1).

### 3.3 Consensus statements for coexistence of NUV and CSU in the same patient

The expert group agreed that CSU and NUV can coexist. Thus, CSU patients may occasionally show signs and symptoms of UV, for example, lesions lasting 24 h or more, which progress to transient purpura or bruising before resolution. Although many core experts agreed that CSU and NUV are part of a disease continuum rather than two different entities, only half of the urticaria specialists approved this consensus (Table S2).

### 3.4 Consensus statements for differential diagnosis between NUV and CSU

**FIGURE 2:**
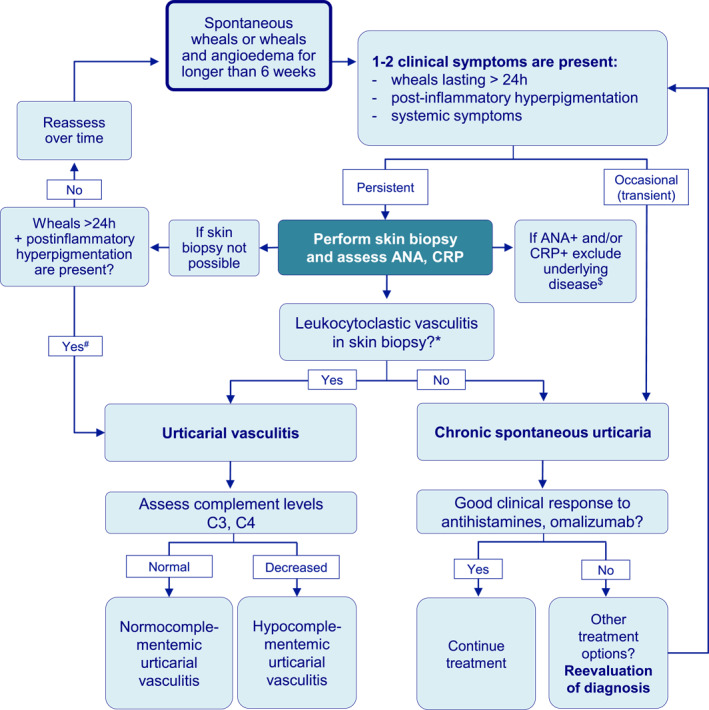
The algorithm of differential diagnosis between urticarial vasculitis and chronic spontaneous urticaria in patients initially presenting with chronic recurrent wheals. ANA, Antinuclear antibodies; CRP, C‐reactive protein. *Leukocytoclasia and fibrin deposits on the walls of the vessels. ^$^Mostly infection and autoimmune diseases, rarely autoinflammatory disease. ^#^Diagnosis of UV is highly probable but must be confirmed by skin biopsy.

The core experts and urticaria specialists agreed that a wheal duration of >24 h and resolution of lesions with residual signs, for example, bruising/postinflammatory hyperpigmentation, as well as systemic symptoms (e.g. abdominal pain, fever, and/or joint pain) should prompt performing lesional skin biopsy in a patient with recurrent wheals. One or two of these clinical findings but not necessarily all three (in the first place bruising/purpura/postinflammatory hyperpigmentation) are considered to be sufficient to recommend a skin biopsy in a patient with recurrent wheals (Figure 2).

It was agreed that the following histologic criteria establish a histopathologic diagnosis of UV: leukocytoclasia and fibrin deposits in the vessel walls. The agreed laboratory criteria in addition to the skin biopsy were anti‐nuclear antibodies (ANA) and C‐reactive protein (CRP) (Table 1).

If skin biopsy cannot be performed for any reason for the differential diagnosis between NUV and CSU, the core expert group and urticaria specialists agreed that long wheal duration (>24 h) and bruising/purpura/postinflammatory hyperpigmentation support the diagnosis of NUV in a patient with recurrent wheals (85/86%), but a skin biopsy is necessary to confirm the diagnosis of NUV (Table 1).

## 4 DISCUSSION

The results of this international EAACI taskforce underline the unmet needs in UV research and propose criteria for the diagnosis of UV and its differential diagnosis with CSU.

In many cases, HUV can be easily differentiated from CSU due to its severe course, systemic symptoms, low levels of complement components C3, C4, and underlying disease,^5^, ^6^ features rarely seen in CSU patients.^2^, ^22^ Therefore, most experts agree that HUV is a different entity and the main diagnostic unmet need is to distinguish CSU from NUV rather than HUV. NUV diagnosis is challenging as indicated by a longer diagnostic delay in NUV versus CSU and HUV patients (median 21 vs. 6 vs. 5 months).^1^, ^10^

There is consensus that the main diagnostic need is to examine whether CSU and NUV are different conditions or a part of an illness spectrum presenting with recurrent wheals. Only 61% of experts manage patients with coexistent CSU and NUV (mostly in <10% of patients). Some experts saw occasional patients progress from one condition to the other (and back again). In the literature, CSU cases with occasional clinical features attributed to UV such as postinflammatory hyperpigmentation have been described,^12^, ^14^ however those cases did not show significant differences in lesional histopathology compared to CSU patients without these features.^23^ In line with this, experts mostly agreed that clinical aspects of transient bruising and/or histology with minor “vascular aggression” (minor leukocytoclasia or red blood cell extravasation) may occur in CSU, particularly during severe episodes, although this is considered to be rare. However, the experts did not agree on the implementation of further diagnostic tests and/or a change of diagnosis in case of occasional occurrence of long‐lasting lesions with transient purpura/bruising in a CSU patient. To sum up, the question of whether CSU and NUV are distinct entities or a part of the disease continuum is still open and should be addressed in further prospective studies with long‐term follow‐up and multiple biopsies.

Skin biopsy and its histopathologic analysis are considered the gold standard for the diagnosis of UV.^2^ However, its relative invasiveness limits the use of skin biopsy, and strict clinical criteria for its indication are needed. The core expert group and urticaria specialists agreed that a wheal duration of >24 h, resolution of lesions with residual signs, for example, bruising/postinflammatory hyperpigmentation, and systemic symptoms (e.g. abdominal pain, fever, and/or joint pain) are the main criteria for performing skin biopsy in a patient with chronic recurrent wheals for differentiating between CSU and UV. The recent UVERSICU study shows that patients with wheals of ≥24 h duration, post‐inflammatory skin hyperpigmentation, and fatigue at disease onset have a 7.3‐, 4.1‐, and 3.1‐fold higher probability of UV diagnosis, respectively.^10^ In the literature, the reported rates of bruising/postinflammatory hyperpigmentation in CSU versus UV patients range from 9% to 21% and 48%–93%, respectively.^1^, ^6^, ^10^, ^14^ Similarly, the reported rates of wheal duration of >24 h in CSU versus UV are 21%–26% versus 63%–94%.^1^, ^10^, ^14^ Experts did not agree on whether lesions appear on the same (69%) or different body sites (23%) in CSU versus UV patients. Several experts assumed that the lower extremities are more often affected by UV but not CSU. No clear difference in wheal distribution between CSU and UV patients was seen in the UVERSICU study using body maps.^10^

Twelve experts specified systemic symptoms as an important diagnostic clue to suspect UV. Fever, arthralgia or arthritis, and malaise or fatigue were reported most frequently (100%, 92%, and 50%, respectively). Among patients with CSU and UV included in the UVERSICU study, 52% and 73%, respectively, had systemic symptoms,^10^ among them joint pain in 18% versus up to 73%, abdominal pain in 12% versus up to 59% and fever in 15% versus up to 49%.^1^, ^6^, ^10^ Lastly, all three features combined, that is, ever occurred wheal duration of >24 h, bruising/postinflammatory hyperpigmentation, and systemic symptoms, were seen in 5% of CSU patients and 37% of UV patients.^10^

An agreed minimum set of histological criteria to establish a histopathologic diagnosis of UV included leukocytoclasia and fibrin deposits on the vessel walls. Both features together with erythrocyte extravasation have been recently proposed as part of a histological diagnosis of UV.^12^ No consensus was reached for the question of which lesions should be biopsied in a patient initially diagnosed with CSU with suspicion of NUV. Here, some experts preferred a new (early) lesion (wheal before bruising, 39%), the non‐bruising area of a lesion that is progressing to bruising (54%), or a late lesion already in the bruising area (54%). Some experts suggested that a second biopsy is needed in some cases for confirmation or clarification of unclear results of the first one.

The agreed laboratory tests to perform in addition to skin biopsy were ANA titers and CRP levels. Rates of ANA + reported previously were 6.2% for CSU and 10.9%–35.5% for UV.^6^, ^24^, ^25^ Although CRP was elevated in a subpopulation of CSU patients and linked to disease activity (31% of patients, mean 0.8 ± 0.7,^12^ median 10.6 mg/L^26^), it was not as high as seen in UV patients (51.2% of patients, mean 3.6 ± 8.9,^12^ median 17.9 mg/L^25^). The increase in one or both parameters in CSU and UV patients can point to an underlying disease, especially autoimmune disease, such as autoimmune thyroid disease, Sjögren's syndrome, or systemic lupus erythematosus.^27^ Nevertheless, up to now, no laboratory tests exist which may differentiate between CSU and UV.

Many physicians worldwide who are not dermatologists do not perform skin biopsies for UV diagnosis.^13^ The reasons for this may include low awareness of urticaria guidelines as well as limited access to skin histopathology expertise.^13^ Core experts/urticaria specialists agreed that if skin biopsy cannot be performed for any reason for differential diagnosis between NUV and CSU, long wheal duration (>24 h) and bruising/purpura/postinflammatory hyperpigmentation (persistent, not occasional) are major criteria for supporting the diagnosis of NUV in a patient with chronic urticarial rash. We recommend performing skin biopsies in any patient with recurrent wheals with suspicion of underlying vasculitis. However, if not possible, clinical criteria can help to avoid delays in proper treatment.

Our study has strengths and limitations. UV is a rare disease and there are a limited number of experts who see these patients worldwide.^13^ We were able to evaluate responses from more than 100 urticaria specialists dealing with CSU and UV patients from different specialized urticaria centers (UCARE) from 40 countries. However, all urticaria specialists are dermatologists and allergists, while UV patients might present, though rarely, to other specialists, for example, rheumatologists. Lastly, almost all core experts are from Europe and six of them have the same affiliation.

This Delphi survey allowed us to establish criteria for the diagnosis and differential diagnosis of UV in patients with recurrent wheals. This can facilitate the diagnostic approach (e.g. help to identify candidates for skin biopsy) and prompt earlier treatment. There are several unmet needs which should be addressed by further studies and initiatives. First, UV‐specific parameters should be implemented in the Chronic Urticaria Registry (CURE).^28^ Next, criteria for distinguishing NUV from neutrophilic urticarial dermatosis^29^ should be developed, including the assessment of intensity and interstitial distribution as well as the role and relevance of the neutrophilic infiltrate. An additional consensus on UV histopathologic features might help to address the bias associated with different time points (early vs. late lesion) and localization (e.g. lower legs) of biopsies. Furthermore, direct immunofluorescence on the skin biopsy can show deposition of complement, fibrin, and immunoglobulins in the blood vessels and/or along the basement membrane zone and its value should be further assessed in UV. Finally, reliable biomarkers and validated disease activity tools for UV are urgently needed.^30^

## AUTHOR CONTRIBUTIONS

**Karoline Krause**: Investigation, data curation, methodology, formal analysis, project administration, writing – original draft. **Hanna Bonnekoh**: Investigation, data curation, formal analysis, project administration, writing – original draft. **Jannis Jelden‐Thurm**: Data curation, formal analysis, project administration, writing – original draft. **Riccardo Asero**: Investigation, writing – review and editing. **Ana Maria Gimenez‐Arnau**: Investigation, writing – review and editing. **José C. Cardoso**: Investigation, writing – review and editing. **Clive Grattan**: Investigation, writing – review and editing. **Emek Kocatürk**: Investigation, writing – review and editing. **Undine Lippert**: Investigation, writing – review and editing. **Marcus Maurer**: Investigation, writing – review and editing. **Martin Metz**: Investigation, writing – review and editing. **Petra Staubach**: Investigation, writing – review and editing. **Margarida Goncalo**: Investigation, data curation, methodology, project administration, writing – original draft. **Pavel Kolkhir**: Investigation, data curation, formal analysis, project administration, writing – original draft.

## CONFLICT OF INTEREST STATEMENT

KK is or recently was an advisor for and/or received research funding from Berlin Chemie, Novartis and Takeda outside the submitted work. HB received honoraria (advisor, speaker) from AbbVie, Intercept Pharma, Novartis, Sanofi‐Aventis and Valenza Bio Inc. outside of the submitted work. JJT has no conflicts of interest to declare. RA has no conflicts of interest to declare. AMG Almirall, Amgen, AstraZeneca, Avene, Celldex, Escient Pharmaceuticals, Genentech, GSK, Instituto Carlos III‐ FEDER, Leo Pharma, Menarini, Novartis, Sanofi–Regeneron, Thermo Fisher Scientific, Uriach Pharma/Neucor. JC has no conflicts of interest to declare. CG Consultancies for Celltrion and Blueprint Medicine, advisory board for Sanofi. EK is or recently was a speaker and/or advisor for and/or has received research funding from Novartis, Menarini, LaRoche Posey, Sanofi, Bayer. UL has been an advisor, speaker or investigator (received grants and/or participated in clinical trials) for AbbVie, ALK‐Abéllo, CSL Behring, Leo Pharma, Meda Pharma, Novartis, Janssen‐Cilag, Sanofi–Regeneron and Shire/Takeda. MMa is or recently was a speaker and/or advisor for and/or has received research funding from Allakos, Allerdia, Amgen, Aralez, ArgenX, AstraZeneca, Celldex, Centogene, CSL Behring, FAES, Genentech, GIInnovation, Innate Pharma, Kyowa Kirin, Leo Pharma, Lilly, Menarini, Moxie, Novartis, Pfizer, Roche, Sanofi/Regeneron, Third HarmonicBio, UCB, and Uriach. MMe has no conflict of interest to declare. PS participated in advisory boards and/or lectures for Abbvie, Almirall, Allergika, Amgen, Beiersdorf, Biocryst, BMS, CSL, Janssen, Klinge, Leo, Lilly, L'Oreal, Novartis, Pfizer, Sanofi, Takeda, and UCB outside the scope of this work. MG participated in advisory boards and/or lectures for Abbvie, Astra‐Zeneca, Leo, Lilly, Novartis, Pfizer, Sanofi, and Takeda, outside the scope of this work. PK was a speaker/consultant for Novartis, ValenzaBio and Roche outside submitted work.

## Supporting information

Figure S1

Table S2

## Data Availability

The data that support the findings of this study are available from the corresponding author upon reasonable request.
