# Supplementary material for: Differential diagnosis between urticarial vasculitis and chronic spontaneous urticaria: An international Delphi survey
Source: Clin Transl Allergy. 2023 Oct 19;13(10):e12305. doi: 10.1002/clt2.12305 (PMC10587388; doi:10.1002/clt2.12305)
Supplement: Supplementary file 1 — Figure S1 [file CLT2-13-e12305-s001.docx]

**Figure S1:** Survey Round 1

**1. Do you agree that defining the diagnostic limits between chronic spontaneous urticaria (CSU) and urticarial vasculitis (UV) is an unmet need?**

Yes

No

If your answer is No, please comment here:

**2. Do you agree with the existent definition of UV that is wheals or erythematous plaques persisting for >24h combined with the histopathologic findings of leukocytoclastic vasculitis?**

Yes

No

If your answer is No, please provide the reasons and include your definition here:

**3. Do you agree that hypocomplementemic UV (HUV) (mostly anti-C1q vasculitis with systemic symptoms and histology of leukocytoclastic vasculitis of postcapillary venules with immunoglobulin deposits) is a different entity with no overlap with CSU?**

Yes

No

If your answer is No, please comment here:

**4. Do you agree that the main diagnostic unmet need is to distinguish CSU from normocomplementemic UV (and not from hypocomplementemic UV)?**

Yes

No

If your answer is No, please comment here:

**5. Do you consider that clinical aspects of transient bruising and/or histology with minor “vascular aggression“ (minor red blood cell extravasation, leukocytoclasia or red blood cell extravasation) may occur in CSU?**

Yes

No

Further comments

**6. Do some of your patients with a typical clinical diagnosis of CSU, occasionally have (particularly during severe episodes) lesions lasting 24h or more, which progress to transient purpura or bruising before resolution?**

Yes

No

In how many of your CSU patients does it occur (in %)?

Further comments

7. **Would the occasional occurrence of long-lasting lesions with transient purpura/bruising in a CSU patient implement further diagnostic tests and/or change your diagnosis? (Please mark all that apply)**

No further diagnostic tests needed

Perform skin biopsy

Maintain the diagnosis of CSU

Change the diagnosis to NUV

Build the diagnosis on additional laboratory results

Build the diagnosis on skin biopsy results

Further comments

**8. Do you manage patients with coexistent CSU and NUV?**

Yes

In how many of your CSU patients does it occur (in %)?

No

Further comments

**9. Which of the following diagnostic criteria do you consider to be required as a minimum set of criteria to establish a histopathologic diagnosis of UV? Please mark all those that you consider essential for the diagnosis of UV.**

a. Leukocytoclasia

b. Erythrocyte extravasation

c. Fibrin deposits

d. Perivascular inflammatory cell infiltrate

e. Endothelial cell swelling

**10. Which of the following diagnostic criteria may - in your opinion – be occasionally observed in CSU as well?**

a. Leukocytoclasia

b. Erythrocyte extravasation

c. Fibrin deposits

d. Perivascular inflammatory cell infiltrate

e. Endothelial cell swelling

**11. Do you see any further unmet needs in the diagnostic approach of differentiating CSU from NUV?**

Please comment:

**Figure S2:** Survey Round 2

**1. Do you agree that the main diagnostic unmet need is to examine whether CSU and normocomplementemic UV are different entities or part of a disease spectrum presenting with wheals?**

Yes

No

**2. Do you think/ observe that lesions appear on same or different body sites in CSU versus UV patients?**

Same

Different

No

If your answer is "different", which body sites do you see in which disease? Please comment:

**3. Do you observe coexistence of CSU and NUV symptoms (e.g. transient wheals and long-lasting lesions with bruising) in patients at the same time?**

Yes

No

If your Answer is “Yes”, in how many of your CSU patients does it occur (in %)?

Further comments:

**4. Do you observe a change in clinical presentation from CSU to NUV and vice versa in some patients over time?**

Yes

No

If your Answer is “Yes”, in how many of your CSU patients does it occur (in %)?

If your answer is "Yes", which symptoms usually appear/disappear?

**5. Based on which clinical findings and/or treatment response do you decide for further diagnostic testing in a CSU patient? Please check all that apply.**

Systemic symptoms

Non-responsiveness to antihistamines

Wheal duration of > 24h

Long disease duration > 1 year

Resolution of lesions with residual hyperpigmentation

Pain or burning of the skin

Severe/active disease

Underlying autoimmune disease

Angioedema

Non-responsiveness to omalizumab

If you chose “systemic symptoms”, which symptoms?

How many of these findings need to be present in order to perform further diagnostic testing? Is one finding enough or a combination of symptoms needed? Please comment.

**6. Which laboratory tests would you perform in addition to skin biopsy in a case of occasional occurrence of long-lasting lesions with transient purpura/bruising in a CSU patient? Please mark all that that apply.**

None

CRP

C3

C4

C1q

D-Dimer

ANA

Blood count

Immunofixation

Ferritin

Serum amyloid A

IgE

Thyroid function test

ASST (autologous serum skin test)

BAT (basophil activation test)

Additional tests:

Further Comments: Please state the diagnostic reason for each test.

**7. If you recommence a skin biopsy in a patient initially diagnosed with CSU with suspicion for NUV, which lesions should be biopsied? Please choose all that apply.**

A new (early) lesion (wheal before bruising)

The non-bruising area of a lesion that is progressing to bruising

A late lesion already in the bruising area

I do not recommend a skin biopsy

On the non-bruising area of a lesion that is progressing to bruising

Further comments (If you perform a biopsy >1 time, please clarify how many times and which lesions should be biopsied)

**8. Which of the following diagnostic criteria do you consider to be required as a minimum set of criteria to establish a histopathologic diagnosis of UV? Please mark only one those that consider essential for the diagnosis of UV.**

Leukocytoclasia

Erythrocyte extravasation

Fibrin deposits in the walls of the vessels

Perivascular inflammatory cell infiltrate

Endothelial cell swelling

**9. Which minor and major clinical and laboratory criteria are critical in differentiating CSU from NUV? Please check all that applies.**

*Minor criteria*

Wheal duration

Wheals+bruising/purpura

Non-response to antihistamines

Systemic symptoms

Underlying disease

Dermoscopy (purpuric patches)

Histopathology

Inflammatory markers

Other laboratory markers

*Major criteria*

Wheal duration

Wheals+bruising/purpura

Non-response to antihistamines

Systemic symptoms

Underlying disease

Dermoscopy (purpuric patches)

Histopathology

Inflammatory markers

Other laboratory markers

Further comments:

**10. Do you believe that CSU and NUV are part of a disease continuum rather than two different entities?**

Yes

No

Further comments:

**11. Are further studies necessary to better characterize the difference and similarities in CSU and UV patients?**

Yes

No

Further comments:

**Figure S3:** Survey Round 3

**1. For how many years have you been managing patients with UV/CSU?**

___ years

**2. How many patients with UV do you see per year?**

____patients

**3. Please indicate your specialty:**

Allergist/Immunologist

Dermatologist

General practitioner (primary care)

Pediatrician

Rheumatologist

If you have chosen other, please specify:

**4. Do you think/observe that lesions appear on the same or different body sites in CSU versus UV patients? (In the previous round of survey, no consensus was reached. Therefore, we would like to rephrase and ask the question again.)**

On the same body sites in the majority of patients.

On different body parts in the majority of patients.

Further comments:

**5. In the previous round, systemic symptoms, wheals of >24h duration and bruising/postinflammatory hyperpigmentation were chosen by most experts among clinical findings and/or treatment responses to decide for further diagnostic tests in CSU patients.**

**Are systemic symptoms (e.g. abdominal pain, fever, and/or joint pain), bruising/postinflammatory hyperpigmentation and long wheal duration (>24 h) main criteria for performing a skin biopsy in a CSU patient for differential diagnosis with urticarial vasculitis?**

Yes

No

Further comments:

**5a. If Yes, how many of these findings (systemic symptoms, bruising/postinflammatory hyperpigmentation, wheals of > 24h duration) need to be present in order to perform a skin biopsy in a CSU patient?**

One

Two

All three

**5b. If one or two, which features exactly?**

Systemic symptoms

Bruising/postinflammatory hyperpigmentation

Wheals of > 24h duration

Further comments:

**6. In your opinion, which clinical and laboratory criteria are critical in differentiating CSU from normocomplementemic UV (NUV)? Please grade the items (1 = most important, 9 = least important)**

Wheal >24 h duration

1 2 3 4 5 6 7 8 9

Bruising/postinflammatory hyperpigmentation

1 2 3 4 5 6 7 8 9

Non-response to antihistamines

1 2 3 4 5 6 7 8 9

Systemic symptoms (e.g. fever, malaise, headache, fatigue, arthralgia, arthritis)

1 2 3 4 5 6 7 8 9

Underlying disease (e.g. infection, autoimmune disorder, cancer)

1 2 3 4 5 6 7 8 9

Dermoscopy (purpuric patches)

1 2 3 4 5 6 7 8 9

Histopathology (signs of leukocytoclastic vasculitis)

1 2 3 4 5 6 7 8 9

Inflammatory markers (CRP/ESR)

1 2 3 4 5 6 7 8 9

Other clinical and laboratory markers

1 2 3 4 5 6 7 8 9

If you have chosen " Other clinical and laboratory markers", please specify

Further comments:

**7. In the previous round, most experts reported that some of their patients with a typical clinical diagnosis of CSU, occasionally have lesions lasting 24h or more, which progress to transient purpura or bruising before resolution and/or histology with minor “vascular aggression“.**

**7a. Among CSU patients without histologic but with clinical NUV features, e.g. wheals >24h duration and/or residual hyperpigmentation, how many do you believe show “true” NUV (leukocytoclastic vasculitis) on skin biopsy over time (a disease continuum)?**

0%

<1%

1-10%

11-20%

21-50%

>50%

Further comments:

**7b. Among CSU patients without histologic and clinical NUV features, how many do you believe show “true” NUV (leukocytoclastic vasculitis) on skin biopsy over time (a disease continuum)?**

0%

<1%

1-10%

11-20%

21-50%

>50%

Further comments:

**8. If you believe that same patients can have both CSU and UV at different time points (disease continuum), when do you think NUV can appear in CSU patients? Please choose all that apply**

During high clinical activity of disease

In patients with autoimmune CSU

In CSU patients with underlying autoimmune disease e.g. SLE

In CSU patients with underlying cancer

Other

If you have chosen Other – Please specify

Further comments:

**9. Is a finding of leukocytoclastic vasculitis (LV) on skin biopsy alone enough for a differential diagnosis between NUV and CSU in a patient presenting with chronic urticarial rash or should skin biopsy results be used in combination with other features?**

Skin biopsy finding of LV alone is enough to diagnose NUV

Skin biopsy finding of LV alone is not enough to diagnose NUV

**9a. If skin biopsy alone is not enough for differential diagnosis of CSU and NUV and should be used in combination with other features, which features are these? Please select three the most important features**

Long wheal duration (<24h)

Bruising/purpura/postinflammatory hyperpigmentation

Systemic symptoms (e.g. fever, malaise, headache, fatigue, arthralgia, arthritis)

Underlying disease (e.g. infection, autoimmune disorder, cancer)

Severe disease

Duration of disease > 1 year

Dermoscopy (purpuric patches)

Inflammatory markers (increased levels of CRP/ESR)

Non-response to antihistamines (standard dose)

Non-response to antihistamines (high dose)

Non-response to omalizumab

Non-response to cyclosporine

Other clinical and laboratory markers

If you have chosen " Other clinical and laboratory features", please specify

**10. If skin biopsy cannot be performed for any reason for differential diagnosis between NUV and CSU, what are other major criteria for diagnosis of NUV in a patient with chronic urticarial rash? Please select three the most important features**

Long wheal duration (<24h)

Bruising/purpura/postinflammatory hyperpigmentation

Systemic symptoms (e.g. fever, malaise, headache, fatigue, arthralgia, arthritis)

Underlying disease (e.g. infection, autoimmune disorder, cancer)

Severe disease

Duration of disease > 1 year

Dermoscopy (purpuric patches)

Inflammatory markers (increased levels of CRP/ESR)

Non-response to antihistamines (standard dose)

Non-response to antihistamines (high dose)

Non-response to omalizumab

Non-response to cyclosporine

Other clinical and laboratory markers

**Figure S4:** Survey Round 4

**1. Do you see patients with urticarial vasculitis (UV)? (If no, please do not complete this survey)**

Yes

No

**2. For how many years have you been managing patients with UV/CSU?**

___ years

**3. How many patients with UV do you see per year?**

____patients

**4. Please indicate your main specialty:**

Allergist/Immunologist

Dermatologist

General practitioner (primary care)

Pediatrician

Rheumatologist

Other, please specify

**5. Are you a member of UCARE?**

Yes

No

My center is currently applying

**6. Please indicate the name of your UCARE**

**7. Your UCARE is missing? Please indicate your UCARE**

**8. The definition of the diagnostic limits between chronic spontaneous urticaria (CSU) and urticarial vasculitis (UV) is an unmet need.**

I agree

I disagree

**9. The existent definition of UV is wheals or erythematous plaques persisting for >24h combined with the histopathologic findings of leukocytoclastic vasculitis.**

I agree

I disagree

**10. Hypocomplementemic UV (HUV) is a different entity with no overlap with CSU.**

I agree

I disagree

**11. The main diagnostic unmet need is to distinguish CSU from normocomplementemic UV (NUV).**

I agree

I disagree

**12. The main diagnostic unmet need is to examine whether CSU and NUV are different entities or part of a disease spectrum presenting with wheals.**

I agree

I disagree

**13. Further studies are necessary to better characterize the difference and similarities in CSU and UV patients.**

I agree

I disagree

**14. Lesions appear on the same body sites in the majority of CSU versus UV patients.**

I agree

I disagree

**15. Clinical aspects of transient bruising and/or histology with minor “vascular aggression“ (minor red blood cell extravasation, leukocytoclasia or red blood cell extravasation) may occur in CSU.**

I agree

I disagree

**16. Some of the patients with a typical clinical diagnosis of CSU, occasionally have (particularly during severe episodes) lesions lasting 24h or more, which progress to transient purpura or bruising before resolution.**

I agree

I disagree

**17. There is the coexistence of CSU and NUV symptoms (e.g. transient wheals and long-lasting lesions with bruising) in some patients at the same time.**

I agree

I disagree

**18. There is a change in clinical presentation from CSU to NUV and vice versa in some patients over time.**

I agree

I disagree

**19. CSU and NUV are a part of a disease continuum rather than two different entities.**

I agree

I disagree

**20. Systemic symptoms, wheal duration of >24h, and resolution of lesions with residual signs, e.g. bruising, are the most important features for further diagnostic testing in a CSU patient.**

I agree

I disagree

**21. Systemic symptoms (e.g. abdominal pain, fever, and/or joint pain), bruising/postinflammatory hyperpigmentation, and long wheal duration (>24 h) are the main criteria for performing a skin biopsy in a CSU patient for differential diagnosis with urticarial vasculitis.**

I agree

I disagree

**22. One or two of these findings but not all three (systemic symptoms, bruising/postinflammatory hyperpigmentation and/or long wheal duration) need to present to perform a skin biopsy in a CSU patient for differential diagnosis with urticarial vasculitis.**

I agree

I disagree

**23. Among these three clinical features (systemic symptoms, bruising/postinflammatory hyperpigmentation and/or long wheal duration), the presence of bruising/postinflammatory hyperpigmentation is more important to perform a skin biopsy in a CSU patient.**

I agree

I disagree

**24. ANA and CRP are laboratory tests performed in addition to skin biopsy in a case of occasional occurrence of long-lasting lesions with transient purpura/bruising in a CSU patient.**

I agree

I disagree

**25. Leukocytoclasia and fibrin deposits on the walls of the vessels are required as a minimum set of criteria to establish a histopathologic diagnosis of UV.**

I agree

I disagree

**26. Among clinical and laboratory criteria (clinical criteria: wheal duration, bruising/purpura, non-response to antihistamines, systemic symptoms, underlying disease; laboratory criteria: dermoscopy, histopathology, inflammatory markers, other laboratory markers), most important major criterion in differentiating CSU from NUV is wheals with bruising/purpura.**

I agree

I disagree

**27. Among clinical and laboratory criteria (clinical criteria: wheal duration, bruising/purpura, non-response to antihistamines, systemic symptoms, underlying disease; laboratory criteria: dermoscopy, histopathology, inflammatory markers, other laboratory markers), most important minor criterion in differentiating CSU from NUV ist dermoscopy.**

I agree

I disagree

**28. Among CSU patients without histologic and clinical NUV features, ≤20% of patients show “true” NUV (leukocytoclastic vasculitis) on skin biopsy over time (a disease continuum).**

I agree

I disagree

**29. If skin biopsy cannot be performed for any reason for differential diagnosis between NUV and CSU, long wheal duration (>24h) and bruising/purpura/postinflammatory hyperpigmentation are major criteria for diagnosis of NUV in a patient with chronic urticarial rash.**

I agree

I disagree

**30. Thank you very much for your time and responses. Do you agree to be acknowledged in the manuscript?**

**If yes, please provide your name:**

**Please provide your email if you would like to participate in further UV-related projects:**
