# Supplementary material for: Differential diagnosis between urticarial vasculitis and chronic spontaneous urticaria: An international Delphi survey
Source: Clin Transl Allergy. 2023 Oct 19;13(10):e12305. doi: 10.1002/clt2.12305 (PMC10587388; doi:10.1002/clt2.12305)
Supplement: Supplementary file 2 — Table S2 [file CLT2-13-e12305-s002.docx]

**Table S1. Characteristics of study participants**

|  | | **Experts**  (n=13) | **Urticaria specialists**  (n=130) |
| --- | --- | --- | --- |
| **Years of experience in managing patients with UV/CSU, median (IQR)** | | 20.0 (15.0-28.0) | 15.0 (10.0-25.0) |
| **Number of UV patients per year treated, median (IQR)** | | 9.0 (4.0-13.5) | 7.5 (3.0-11.5) |
| **Expertise, % (n)** | Dermatology | 76.9 (10) | 43.8 (57) |
|  | Allergology/clinical immunology | 23.1 (3) | 56.2 (73) |
| **Region, % (n)** | **Asia** (China, India, Thailand, Georgia, Japan, Korea, Russia, Philippines, Kazakhstan) | 0.0 (0) | 13.9 (18) |
|  | **Australasia** (Australia) | 0.0 (0) | 1.5 (2) |
|  | **Africa and Middle East** (Turkey, Qatar, Oman, Iran, Saudi Arabia, South Africa, United Arab Emirates) | 8.0 (1) | 13.9 (18) |
|  | **Americas** (Brazil, USA, Argentina, Canada, Peru, Columbia, Ecuador, Mexico) | 0.0 (0) | 23.8 (31) |
|  | **Europe** (Germany, France, Poland, Spain, UK, Bulgaria, Greece, Austria, Denmark, Netherlands, Ireland, Italy, Belgium, North Macedonia, Portugal, Slovenia, Romania, Switzerland) | 92.0 (12) | 46.9 (61) |

UCARE, Urticaria Centers of Reference and Excellence; UV, Urticarial vasculitis; CSU, Chronic spontaneous urticaria; IQR, interquartile range

**Table S2. Summary of all statements (with and without full consensus) after the final fourth round of the Delphi survey** (% agreement core experts/urticaria specialists)*

| **Unmet needs in differential diagnosis between UV and CSU** |
| --- |
| - The definition of the diagnostic limits between chronic spontaneous urticaria (CSU) and urticarial vasculitis (UV) is an unmet need (100/88%) - The existent definition of UV is wheals or erythematous plaques persisting for >24h combined with the histopathologic findings of leukocytoclastic vasculitis (93/96%) - Hypocomplementemic UV (HUV)** is a different entity with no overlap with CSU (85/80%) - The main diagnostic unmet need is to distinguish CSU from normocomplementemic UV (and not from HUV) (85/86%) - The main diagnostic unmet need is to examine whether CSU and NUV are different entities or part of a disease spectrum presenting with wheals (100/94%) - Further studies are necessary to better characterize the difference and similarities in CSU and UV patients (100/99%) |
| **Coexistence of UV/NUV and CSU in same patients** |
| - Lesions appear on the same body sites in the majority of CSU versus UV patients (100/66%) - Clinical aspects of transient bruising and/or histology with minor “vascular aggression“ (minor red blood cell extravasation, leukocytoclasia or red blood cell extravasation) may occur in CSU (92/78%) - Some of patients with a typical clinical diagnosis of CSU, occasionally have (particularly during severe episodes) lesions lasting 24h or more, which progress to transient purpura or bruising before resolution (92/81%) - There is coexistence of CSU and NUV symptoms (e.g. transient wheals and long-lasting lesions with bruising) in patients at the same time (100/92%) - There is a change in clinical presentation from CSU to NUV and vice versa in some patients over time (85/74%) - CSU and NUV are part of a disease continuum rather than two different entities (85/50%) |
| **Clinical and laboratory criteria for differential diagnosis** |
| - Long wheal duration (>24 h), bruising/postinflammatory hyperpigmentation, and systemic symptoms (e.g. abdominal pain, fever, and/or joint pain) and are the main criteria for performing a skin biopsy in a CSU patient for differential diagnosis with urticarial vasculitis (100/92%) - One or two of these findings but not necessarily all three (systemic symptoms, bruising/postinflammatory hyperpigmentation and/or long wheal duration) are enough to perform a skin biopsy in a CSU patient for differential diagnosis with urticarial vasculitis (100/87%) - Leukocytoclasia and fibrin deposits in the walls of the vessels are required as a minimum set of criteria to establish a histopathologic diagnosis of UV (92/93%) - If skin biopsy cannot be performed for any reason for differential diagnosis between NUV and CSU, long wheal duration (>24h) and bruising/purpura/postinflammatory hyperpigmentation are major criteria for possible diagnosis of NUV in a patient with recurrent wheals (85/86%) - Systemic symptoms (100%), wheal duration of >24h (92%) and resolution of lesions with residual signs, e.g. bruising (92%) were most frequently selected responses for further diagnostic testing in a CSU patient (other specialists 83%) - ANA and CRP are laboratory tests that should be performed in addition to biopsy in a case of occasional occurrence of long-lasting lesions with transient purpura/bruising in a CSU patient (92/91%) - Major and minor clinical and laboratory criteria critical in differentiating CSU from NUV include wheals with bruising/purpura (major criterion) (92/73%) and dermoscopy (minor criterion) (85/44%) - Lesions appear on the same body sites in the majority of CSU versus UV patients (100/66%) - Among these three clinical features (systemic symptoms, bruising/postinflammatory hyperpigmentation and/or long wheal duration), the presence of bruising/postinflammatory hyperpigmentation is more important to perform a skin biopsy in a CSU patient (85/75%) - Among CSU patients without histologic and clinical NUV features, ≤20% of patients show “true” NUV (leukocytoclastic vasculitis) on skin biopsy over time (a disease continuum) (92/75%) |

*Based on the three rounds of Delphi survey of 13 experts, all coauthors of this publication, 22 statements have been prepared with consensus 85% or more. 130 urticaria specialists, dermatologists and allergists, most from the Urticaria Centers of Reference and Excellence (UCARE) worldwide, were asked to agree or disagree the consensus statements. Statements that did not reach consensus (<85%) for core experts but not urticaria specialists are not shown. Although no consensus was reached among the experts, 62% suggested performing a skin biopsy in CSU patients with occasional occurrence of long-lasting purpura with transient bruising while 62% of experts would collect additional laboratory results first. Most frequently recommended laboratory tests were CRP (92%), ANA (92%), C4 (77%), blood count (69%), C3 (62%) and C1q (54%), while 62% of experts indicated that histological evidence of leukocytoclastic vasculitis alone is enough to diagnose NUV in a patient with recurrent wheals. If skin biopsy is necessarily combined with other features to diagnose CSU or UV, most experts additionally preferred the presence of clinical symptoms such as wheals lasting up to 24 hours (75%), postinflammatory hyperpigmentation (75%) or systemic symptoms (75%). Many experts also stated that in patients with both CSU and NUV at different time points, NUV appears especially during high clinical activity of CSU (77%) or in CSU with underlying autoimmune disease (62%), without reaching consensus.

**mostly anti-C1q vasculitis with systemic symptoms and histology of leukocytoclastic vasculitis of postcapillary venules with immunoglobulin deposits
